# Imaging an aligned polyatomic molecule with laser-induced electron diffraction

**DOI:** 10.1038/ncomms8262

**Published:** 2015-06-24

**Authors:** Michael G. Pullen, Benjamin Wolter, Anh-Thu Le, Matthias Baudisch, Michaël Hemmer, Arne Senftleben, Claus Dieter Schröter, Joachim Ullrich, Robert Moshammer, C. D. Lin, Jens Biegert

**Affiliations:** 1 ICFO-Institut de Ciencies Fotoniques, Mediterranean Technology Park, Castelldefels (Barcelona) 08860, Spain; 2 J. R. Macdonald Laboratory, Department of Physics, Kansas State University, Manhattan, Kansas 66506-2604, USA; 3 Universität Kassel, Institut für Physik und CINSaT, Heinrich-Plett-Strasse 40, Kassel 34132, Germany; 4 Max-Planck-Institut für Kernphysik, Saupfercheckweg 1, Heidelberg 69117, Germany; 5 Physikalisch-Technische Bundesanstalt (PTB), Bundesallee 100, Braunschweig 38116, Germany; 6 Department of Physics and Astronomy, University of New Mexico, 1919 Lomas Boulevard NE, Albuquerque, New Mexico 87131, USA; 7 ICREA-Institució Catalana de Recerca i Estudis Avançats, Barcelona 08010, Spain

## Abstract

Laser-induced electron diffraction is an evolving tabletop method that aims to image ultrafast structural changes in gas-phase polyatomic molecules with sub-Ångström spatial and femtosecond temporal resolutions. Here we demonstrate the retrieval of multiple bond lengths from a polyatomic molecule by simultaneously measuring the C–C and C–H bond lengths in aligned acetylene. Our approach takes the method beyond the hitherto achieved imaging of simple diatomic molecules and is based on the combination of a 160 kHz mid-infrared few-cycle laser source with full three-dimensional electron–ion coincidence detection. Our technique provides an accessible and robust route towards imaging ultrafast processes in complex gas-phase molecules with atto- to femto-second temporal resolution.

## Abstract (web summary)

Laser-induced electron diffraction can provide structural information on gas-phase molecules with high spatial and temporal resolution. Going beyond previous diatomic cases, Pullen *et al.* apply this approach to acetylene and show that it can be used to measure bond lengths for polyatomic molecules.

## Graphical abstract

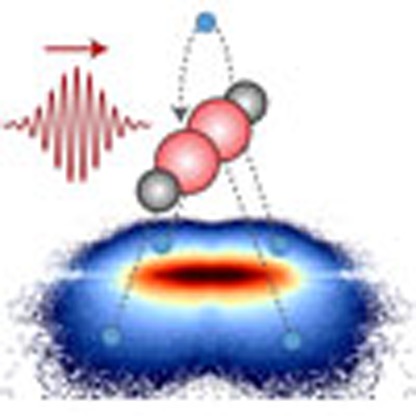

Dynamic imaging of chemical reactions or biological functions is one of the grand challenges of science^1^,^2^. These processes are typically triggered by sub-Ångström-scale events that are initiated on the few-femtosecond timescale. An imaging method that could achieve the required spatiotemporal resolutions would provide revolutionary insights into the connection between molecular structure at critical transition points and barrier heights; hallmark examples are transition states^3^, rapid dynamics in the vicinity of conical intersections^4^ or proton migration and isomerization^5^. The capability of imaging the motions of the atomic constituents during these processes with few-femtosecond temporal and sub-Ångström spatial resolutions therefore represents a paradigm shift in modern physics and chemistry.

Ultrafast electron diffraction (UED) is capable of resolving atomic positions with sub-Ångström resolution^6^, however, the achievable temporal resolution is currently limited to hundreds of femtoseconds mainly due to Coulomb repulsion in the electron bunch. Such temporal resolution is not sufficient to resolve the initiation reactions and ultrafast changes of the prototypical processes mentioned above. Current developments therefore aim at reducing space charge^7^ or using relativistic electron bunches^8^. X-ray diffraction methods^9^ currently suffer from spectrotemporal jitter and are only available at large-scale facilities. These restraints have motivated the development of new dynamical imaging techniques, largely for the gas phase, such as chirped-encoded recollisions^10^, photoelectron holography^11^, femtosecond photoelectron diffraction^12^, Coulomb explosion imaging^13^ and laser-assisted electron diffraction^14^.

Clearly, versatile laboratory-scale tabletop methods that provide the combined spatial and temporal resolutions would signify a breakthrough, especially for the imaging of gas-phase molecular dynamics. Laser-induced electron diffraction (LIED) is such a method and is based on probing an objects structure using its own electrons that are rescattered during strong-field-induced recollisions^15^,^16^,^17^. This process is depicted in Fig. 1 where the longitudinal and transverse momenta are defined as *k*_||_=*k*_y_ and 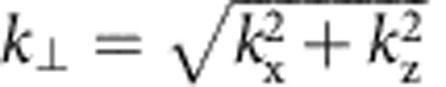, respectively. Coherent subcycle elastic scattering of the electron wavepacket with attosecond (single pulse) to femtosecond (pulse train) resolution retains structural information of the ionic species in the resultant diffraction pattern^18^,^19^,^20^,^21^. The challenge lies in simultaneously fulfilling the extremely stringent conditions for LIED in order to extract structural information; these are as follows: (i) achieving high recollision energies despite a small fraction of target ionization, (ii) achieving core penetrating collisions and sufficient momentum transfer with the scattered electron, (iii) driving recollision in the quasi-static regime to enable extraction of field-free diffraction data from the photoelectron momentum spectra. When these conditions are met, the method of molecular structure retrieval is similar to conventional electron or X-ray diffraction, with the added benefit of femtosecond temporal resolution of the driving laser. In general, state of the art near-infrared lasers cannot fulfil these combined conditions, however, investigations have still been undertaken for homonuclear diatomic molecules such as O_2_ (ref. 19). Recently, these conditions were satisfied with ∼2 μm lasers and structural retrieval of N_2_ and O_2_ molecules was demonstrated^22^,^23^. Spatial resolutions of 0.05 Å were reported, which were sufficient to image a 0.1 Å contraction of the simple O_2_ molecule during the ∼5 fs it takes an electron to rescatter. This result established the potential of LIED as a dynamical imaging technique with sub-Ångström spatial and few-femtosecond temporal resolutions.

To harness the combined temporal and spatial resolutions of LIED and apply it to polyatomic molecules (that is, systems with three or more atoms that exhibit full prototypical molecular dynamics) requires addressing a decisive and unresolved issue, namely the fact that launching the recollision (imaging) electron initiates molecular distortion and eventually fragmentation. Therefore, a certain portion of the detected electrons serves as an unwanted background that can make imaging difficult or even impossible. This problem can be resolved through ion–electron coincidence detection and the retrieval of the doubly differential cross-section. This ensures unambiguous imaging of the molecular structure, or fragments, of interest. In addition to this major concern, there are other experimental obstacles that must be overcome. First, complex molecules commonly have ionization energies around and below 10 eV, which necessitates the use of mid-infrared driving lasers in order to avoid ionization saturation. Mid-infrared sources also have the added benefit that electrons with the required energies are liberated at lower intensities, which results in less distortion of the molecule. Second, because each constituent atom has a unique scattering cross-section, a careful selection of the electron-scattering parameters ensures that they all contribute significantly to the scattering and hence facilitates the simultaneous determination of multiple bond lengths. Third, to resolve the increased structural complexity, it is highly beneficial for the gas ensemble to be anisotropically distributed with respect to the molecular axis in order to remove averaging effects^20^.

Here we meet all of these challenges through a combination of experimental methodologies. A unique home-built optical parametric chirped pulse amplification (OPCPA) source provides 1.7 μm and 3.1 μm pulses at a repetition rate of 160 kHz (ref. 24) and with excellent long-term stability. The 1.7 μm light is used to impulsively align the target molecule, while the 3.1 μm light induces electron rescattering. The lower efficiency of the rescattering process^25^,^26^ at longer wavelengths is more than compensated for by the two orders of magnitude higher repetition rate of our source compared with typical 1 kHz systems. Equally as important is the reaction microscope (ReMi) detection system that allows a careful selection of the relevant channels (over both electron energies and scattering angles) from the doubly differential cross-section in coincidence^27^. We validate our unique experimental approach by simultaneously imaging the C–C and C–H bond lengths of aligned polyatomic molecule acetylene (C_2_H_2_). This establishes LIED as a methodology for dynamically visualizing larger and heteronuclear molecular structures. We chose acetylene as the test molecule since it is heteronuclear, readily alignable, linear and symmetric so that orientation is not required, and its bond lengths are accurately known. More importantly, however, is the fact that acetylene is a prototypical organic molecule in which the dynamics associated with isomerization, proton migration, internal vibrational redistribution of energy and conical intersections can be studied in the future using LIED. The measured bond lengths lie within <5% of the expected acetylene cation equilibrium distances of 1.25 and 1.08 Å (ref. 28), respectively, for both molecular alignments.

## Results

### Extraction of molecular structure

**Figure 2:**
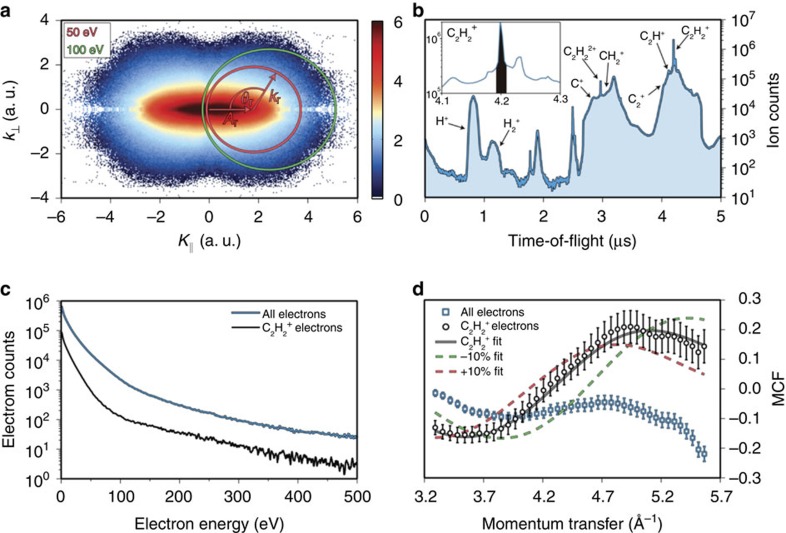
Method to extract structural information from the momentum distributions. (**a**) Logarithmically scaled momentum distribution of electrons corresponding to all ionic fragments. The circles represent the scattering of electrons with the same energy at different angles. (**b**) The detected ion TOF showing the numerous fragments created during the strong-field interaction. The inset shows the peak corresponding to the C_2_H_2_^+^ ion near 4.2 μs and the shaded region represents the window of ions that the C_2_H_2_^+^ electrons are taken from. (**c**) The electron kinetic energy distribution for the C_2_H_2_^+^ ion (black) and for all possible fragmentation processes (blue). (**d**) An extracted MCF for the acetylene cation (black circles) as well as for electrons from all fragments (blue squares). The solid black curve shows the best fit, which matches very well with the cation channel. The MCFs for ±10% changes in the C_2_H_2_ molecular lengths (dashed curves) highlight the sensitivity of the LIED technique. The s.d. error bars are derived from Poissonian statistics.

The procedure for extracting structural information from aligned (see Supplementary Note 1 and Supplementary Figs 1 and 2) C_2_H_2_ using LIED is outlined in Fig. 2. Figure 2a shows the momentum distribution of all electrons detected in coincidence with all positive fragments after the ionization of C_2_H_2_ with our mid-infrared source. Following the quantitative rescattering theory (see Supplementary Note 2), the molecular differential cross-section (DCS) is extracted by sweeping the scattering angle (*θ*_r_) around the circumference of a circle with radius equal to the momentum of the rescattered electron (*k*_r_). The influence of the ionizing laser field must be considered; consequently, the origin of the circle is given by the vector potential (*A*_r_) at the time of rescattering (see the Supplementary Note 3 and Supplementary Fig. 3). Each circle represents rescattering by different electron energies. The extracted experimental molecular DCS (*σ*_M_) is combined with the theoretical atomic DCS (*σ*_A_), which is calculated using the independent atom model (see the Supplementary Note 4), for the same electron energy and emission angle to calculate the molecular contrast factor (MCF) *M*_F_=(*σ*_M_–*σ*_A_)/*σ*_A_. The MCFs are typically presented as a function of the momentum transfer *q*=2*k*_r_ sin(*θ*_r_/2) experienced by the rescattered electrons. A *χ*^2^-based fitting routine is used to compare the experimentally obtained MCF to theoretical predictions (see the Supplementary Note 5 and Supplementary Fig. 4).

### Full-particle coincidence detection in three dimensions

The coincidence detection capability of the ReMi is crucial for accurate retrieval of polyatomic molecular structure from the experimental MCF. To develop the time-resolving capabilities of LIED, it is important that we ensure the scattering pattern originates from the fragmentation channel of interest only. To highlight this point we present the time-of-flight (TOF) spectrum of all the detected positively charged fragments in Fig. 2b. The main peak near 4.2 μs is the acetylene cation (C_2_H_2_^+^) investigated in this manuscript, and it constitutes ∼10% of the total number of detected fragments. The inset shows a close-up of this peak and the black-shaded area represents the region that the electrons associated with C_2_H_2_^+^ are extracted from. Many other fragments can be observed and identified in the TOF and each of these peaks has associated electrons. Figure 2c shows the measured electron kinetic energy spectrum for all fragments (blue) and for the C_2_H_2_^+^ fragment only (black). An order of magnitude difference in the number of detected electrons is visible over the entire spectral range (see Methods and Supplementary Fig. 5). It is these omnipresent extra electrons that serve as an unwanted background signal and are detrimental to structure retrieval without coincidence detection. Figure 2d summarizes this decisive point by comparing the MCFs retrieved when analysing electrons corresponding to all fragments (blue) and from C_2_H_2_^+^ only (black). The C_2_H_2_^+^ data result in an MCF that compares well with the equilibrium acetylene structure, which indicates that, in the case of acetylene, launching the recollision electron does not cause detrimental differences between the neutral and ionic species within the short recollision time. On the other hand, using electrons from all fragmentation channels results in a dramatically different MCF that cannot be accurately fitted (blue squares) and fails in retrieving the C_2_H_2_^+^ bond lengths. We validate with this analysis that electron–ion coincidence detection is a prerequisite for the application of LIED to larger molecules. The high sensitivity of LIED to the exact molecular structure is also illustrated in Fig. 2d by the dramatic change induced in the MCF by a 10% contraction (green) or expansion (red) of the molecule.

### Simultaneous measurement of multiple bond lengths

**Figure 3:**
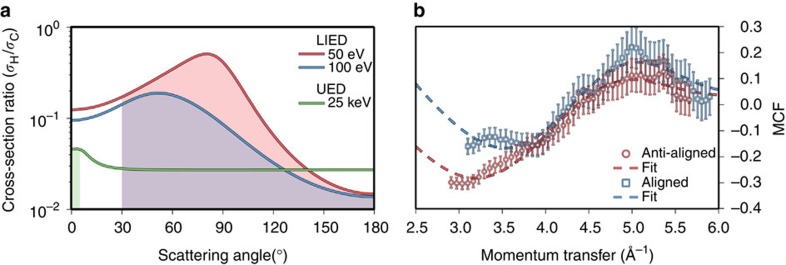
Simultaneous extraction of multiple bond lengths from polyatomic molecules. (**a**) The ratio of the H and C scattering cross-sections as a function of electron-scattering angle for typical energies used in LIED (50 and 100 eV) and CED/UED (25 keV). The ratios are much higher for the energies relevant to LIED and are also applicable over a much wider angular range (shaded regions). (**b**) Blue squares (red circles) show the experimental molecular contrast factor that results from the scattering of 60 eV electrons by aligned (anti-aligned) molecules. The best theoretical fits (dashed lines) allow the accurate extraction of the C–H and C–C bond lengths from both alignments. The s.d. error bars are derived from Poissonian statistics.

To visualize complex molecules, LIED needs to be able to retrieve multiple bond lengths between different atomic species. We demonstrate that our implementation of LIED fulfills this promise and is even able to image the (typically) elusive hydrogen atom by exploiting the fact that we measure the full doubly differential cross-section. At the tens of keV electron energies used in UED, a hydrogen atom has a scattering cross-section (*σ*_H_) that is typically much less than that of a C atom (*σ*_C_). Figure 3a presents the *σ*_H_/*σ*_C_ ratio^29^ for a 25 keV electron as a function of the scattering angle (green curve). The ratio is maximal for scattering angles between 0 and 5° that are typical in UED (shaded region) but even in this range *σ*_H_/*σ*_C_<0.05. The electron energies used in LIED result in much higher values of the *σ*_H_/*σ*_C_ ratio, as is also presented in Fig. 3a for 50 eV (red curve) and 100 eV (blue curve) electrons, because of a minimum in the C atom differential cross-section. Both of the presented energies have wide angular regions where *σ*_H_/*σ*_C_>0.10 and a peak of *σ*_H_/*σ*_C_>0.50 is observed near 80° for 50 eV electrons. The shaded regions represent the much wider scattering angles for which the LIED technique is valid.

To take advantage of the favourable cross-section ratio available to LIED, we confirm that we can simultaneously measure both the C–H and C–C bonds. The MCFs extracted for both molecular alignments after scattering of 60 eV electrons are presented in Fig. 3b. For aligned molecules (blue squares) the best theoretical fit (dashed blue curve) from the *χ*^2^-fitting routine results in bond lengths of 
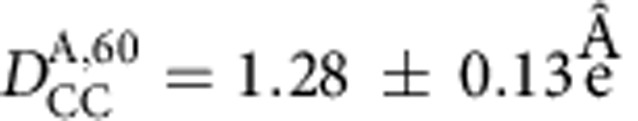
 and 
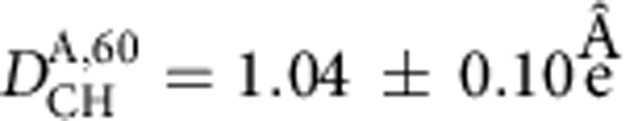
, while for anti-aligned molecules (red circles) the same procedure results in estimates of 
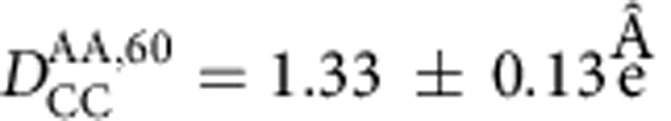
 and 
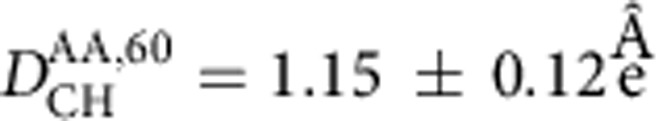
. Here the notation 
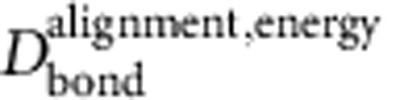
 is used for the results of the individual fits to refer to energy-specific bond lengths. The estimated bond lengths agree well with the known values^28^, and the accuracy of each fit is ∼10 pm, which is an order of magnitude lower than the de Broglie wavelength of the scattering electrons (*λ*_E_=1.3 Å). The positions of the MCF extrema and zero crossings, as well as the peak-to-peak modulation, are very sensitive to changes in the bond lengths and the molecular alignment. It is the sensitivity to these parameters that is utilized to monitor sub-Ångström changes in molecular structure. The two MCFs presented in Fig. 3b show some differences such as the position of the minimum near *q*=3.5 Å^−1^, which is closer to zero for anti-aligned acetylene, and the modulation amplitude, which is smaller in the aligned case. Depending on the target and the degree to which it is aligned, molecular alignment or anti-alignment can lead to larger differences in the peak-to-peak amplitude of the MCFs, which is beneficial for structural imaging. These results confirm that LIED can simultaneously extract multiple bond lengths from complex polyatomic molecules with high accuracy.

### Temporal resolution of LIED

**Figure 4:**
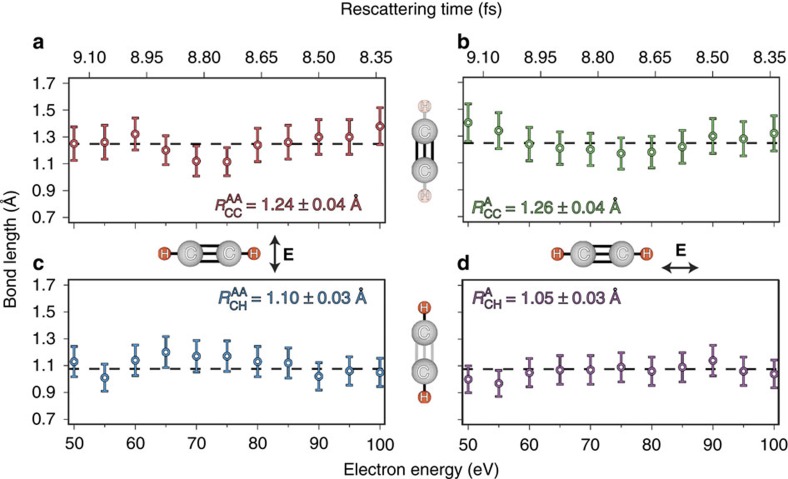
Accurate C_2_H_2_ bond length extraction. The C–C (C–H) bond length estimates are presented as a function of the scattering electron energy and rescattering time in the top (bottom) quadrant. The expected equilibrium values of the acetylene cation are also shown (dashed black lines). The values of the best horizontal fits for each bond are displayed in the respective panels. See Supplementary Fig. 4 for details about the bond length estimate error bars.

Next, we illustrate the possible attosecond temporal resolution^30^ of the technique in Fig. 4. We measure the doubly differential cross-section, which permits retrieving the C–C and C–H bond lengths as a function of the rescattering electron energy. On the basis of operating mid-infrared LIED in the quasi-static limit we can invoke the classical rescattering model to associate a specific time to the measured electron-rescattering energy. The top axis in Fig. 4 shows the corresponding return time for each electron energy and indicates that a temporal resolution below 100 as could be achieved by analysing at different rescattering energies. We further elaborate that the measured energy range can also be used to establish an unprecedented level of confidence and redundancy for the retrieved bond length. The extracted 
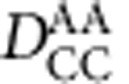
, 
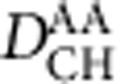
, 
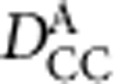
 and 
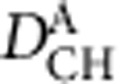
 values are consistent with the estimated ionic equilibrium values (dashed black lines)^28^ over the investigated energy range. As no significant structural rearrangements are expected after acetylene is ionized from a neutral to a cation^28^, fitting a horizontal line to the energy-dependent bond length estimates will yield an overall estimate of the C–C and C–H bond lengths. This fitting results in estimates of 
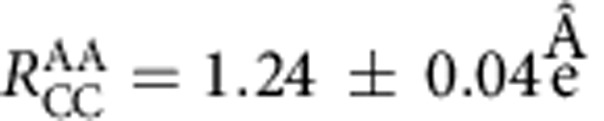
 and 
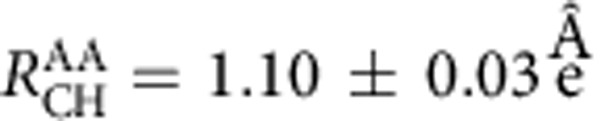
 for anti-aligned molecules while the same analysis with aligned molecules results in bond lengths of 
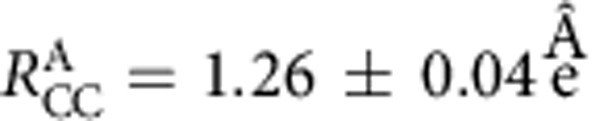
 and 
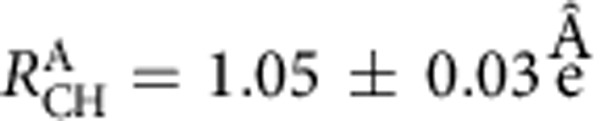
. This method amounts to performing two-dimensional fitting over both electron energy and scattering angle, which is not possible with other techniques, and highlights the accuracy of the LIED method.

## Discussion

In summary, we demonstrate a robust method for the retrieval of multiple bond lengths from an aligned polyatomic molecule with mid-infrared LIED, which we validate by accurately determining the structure of acetylene. The use of a ReMi in combination with a home-built 160 kHz mid-infrared OPCPA exploits coincidence detection together with the measurement of the doubly differential elastic scattering cross-section in the quasi-static regime. This unique capability enables imaging the hydrogen atom by selection of a suitable scattering energy range for which the relative cross-sections contribute comparably. An excellent bond length confidence level is achieved due to the large range of rescattering energies for which structural information is measured. Our method demonstrates a clear path to exploit the intrinsic atto- to femto-second temporal resolution of LIED for the imaging of complex molecules. Finally, we provide a solution to selective imaging of the multiple fragmentation pathways that are inherently created when launching the recollision electron in polyatomic molecules. This capability is an enabling step towards time-resolved imaging and permits accurate retrieval of the geometrical structure from the fragment of interest only. Our data already contain structural information on the ultrafast isomerization and deprotonation of acetylene and on multiply charged ions, which we aim to investigate in future work. The technique provides an accessible and robust route towards probing ultrafast processes in complex gas-phase molecules by combining attosecond and collision physics towards realising the molecular movie.

## Methods

### Mid-infrared OPCPA source

The OPCPA-based source used in this work has been presented previously^24^. It provides 6.5-cycle (70 fs) mid-infrared pulses at a repetition rate of 160 kHz. The 3.1 μm radiation is derived from difference frequency generation of 1.55 and 1.05 μm pulses in a magnesium oxide doped periodically poled lithium niobate (MgO:PPLN) crystal. It is subsequently chirped and parametrically amplified in four cascaded OPA stages before the pulse is compressed to 70 fs in a grating compressor. After focussing with a 50 mm parabolic mirror an estimated peak intensity of 5.5 × 10^13^ W cm^−2^ was reached. This corresponds to a ponderomotive energy of *U*_P_=50 eV and a Keldysh parameter of *γ*=0.34 for C_2_H_2_, which has an ionization potential of 11.4 eV. Owing to the quadratic scaling of the maximum rescattering electron energy with laser wavelength (*E*_max_∝*λ*^2^), our laser can generate much more energetic electrons compared with a ubiquitous 800nm Ti:Sapphire laser. These electrons are a basic requirement for LIED as they penetrate deep into the core of the molecule, thereby revealing structural information. The low mid-infrared photon energy also ensures that complex molecular targets, which typically have low ionization energies, are not in the ionization saturation regime for the required high intensities. The high 160 kHz repetition rate ensures a high data accumulation rate and more than compensates for the lower mid-infrared rescattering probability compared with the typical 800nm, 1 kHz systems. The phase-coherent signal output of the OPCPA at 1.7 μm is utilized to induce impulsive molecular alignment, as is discussed below. This radiation has a pulse duration of ∼98 fs and also operates at a repetition rate of 160 kHz. Both outputs have high stability with power fluctuations less than a percent being typical over the course of the data acquisition period.

### Reaction microscope detection system

We utilize a ReMi detection system to detect the high-energy rescattered electrons. For a thorough overview of the function and capabilities of ReMis see ref. 27. A cold and thin molecular jet is formed by supersonically expanding gas into vacuum and subsequent skimming. The gas is ionized in the interaction region and the resultant charged particles are guided towards opposing position-sensitive microchannel plate detectors by homogenous electric (**E**) and magnetic (**B**) fields. Momentum distributions of both ions and electrons are then extracted from the position and time of detection. To detect high-energy electrons in three dimensions, fields of **E**=51 V cm^−1^ and **B**=39 G are chosen. The scaling of momentum resolution (∂*k*) with field strengths has been discussed previously^27^, and our calculations show that the momentum resolutions are comparable to the momentum integration ranges (Δ*k*) used to obtain the angularly resolved DCSs (see Supplementary Fig. 3 for more information), that is, ∂*k*≈Δ*k*. For example, a 50 eV electron-scattering at an angle of *θ*_r_=50° has a calculated momentum resolution of ∼∂*k*=±15.3%, which is almost the same as the integration range of Δ*k*=±15% used at that point. Therefore, the detection resolution limit of the ReMi is not a limiting factor to this work.

ReMis possess a number of decisive benefits over typical TOF spectrometers for LIED experiments, such as the direct extraction of the full three-dimensional electron momentum distribution and the capability of detecting electrons in coincidence with ions, which allows a simple removal of unwanted electron counts in post-processing. Figure 2c in the main text presents electron energy spectra for electrons associated with all of the detected ionic fragments (blue) and for electrons associated with the C_2_H_2_^+^ ion only (black). A large difference between the two curves can be observed for the entire spectral range. It is important not to include these unwanted electrons (which originate from different ionization and fragmentation processes) in the data analysis as they interfere with bond length determination.

### Molecular fragmentation

In Supplementary Fig. 5a,b we present the measured ionic TOFs for the simple diatomic molecule O_2_ and for the polyatomic molecule C_2_H_2_, respectively. The O_2_ TOF shows a clear lack of fragments with only the single and double ions significantly contributing, while the C_2_H_2_ TOF is full of other ionic fragments. In Supplementary Fig. 5c,d the electrons corresponding to the main single ion (black curves) for each TOF are present along with the electrons corresponding to all ionic fragments (coloured curves). An order of magnitude difference is observed over the entire spectrum in the case of C_2_H_2_, while in the O_2_ case the single ion electrons make up >80% of those detected. This is the reason that electron–ion coincidence detection apparatuses are required to perform LIED on polyatomic molecules.

## Additional information

**How to cite this article:** Pullen, M. G. *et al.* Imaging an aligned polyatomic molecule with laser-induced electron diffraction. *Nat. Commun.* 6:7262 doi: 10.1038/ncomms8262 (2015).

## Supplementary Material

Supplementary Information Supplementary Figures 1-5, Supplementary Notes 1-5 and Supplementary References

## Figures and Tables

**Figure 1:**
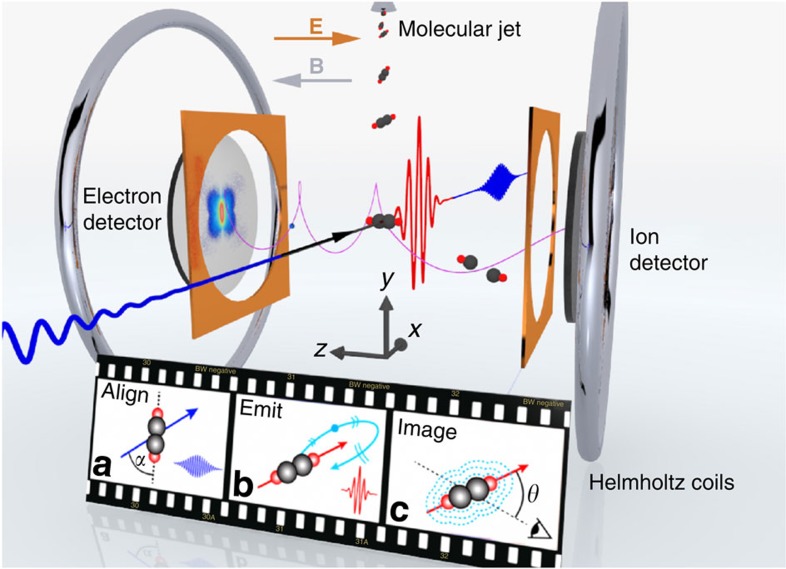
Laser-induced electron diffraction from aligned C_2_H_2_ molecules using a mid-infrared OPCPA source and a reaction microscope. The cartoon film shows the procedure. (**a**) The C_2_H_2_ molecules are pre-aligned by focusing the 1.7 μm pump pulse (blue) into a molecular jet. (**b**) The 3.1 μm pulse (red) is used to generate high-energy electrons that subsequently rescatter off the parent ion. (**c**) The rescattered electrons carry structural information of the parent ion that is contained in the detected angular momentum distributions. The anticollinear electric (**E**) and magnetic (**B**) fields guide the charged fragments towards opposing position-sensitive detectors.
