# Supplementary material for: Imaging an aligned polyatomic molecule with laser-induced electron diffraction
Source: Nat Commun. 2015 Jun 24;6:7262. doi: 10.1038/ncomms8262 (PMC4491169; doi:10.1038/ncomms8262)
Supplement: Supplementary Information — Supplementary Figures 1-5, Supplementary Notes 1-5 and Supplementary References [file ncomms8262-s1.pdf]

Supplementary Figures

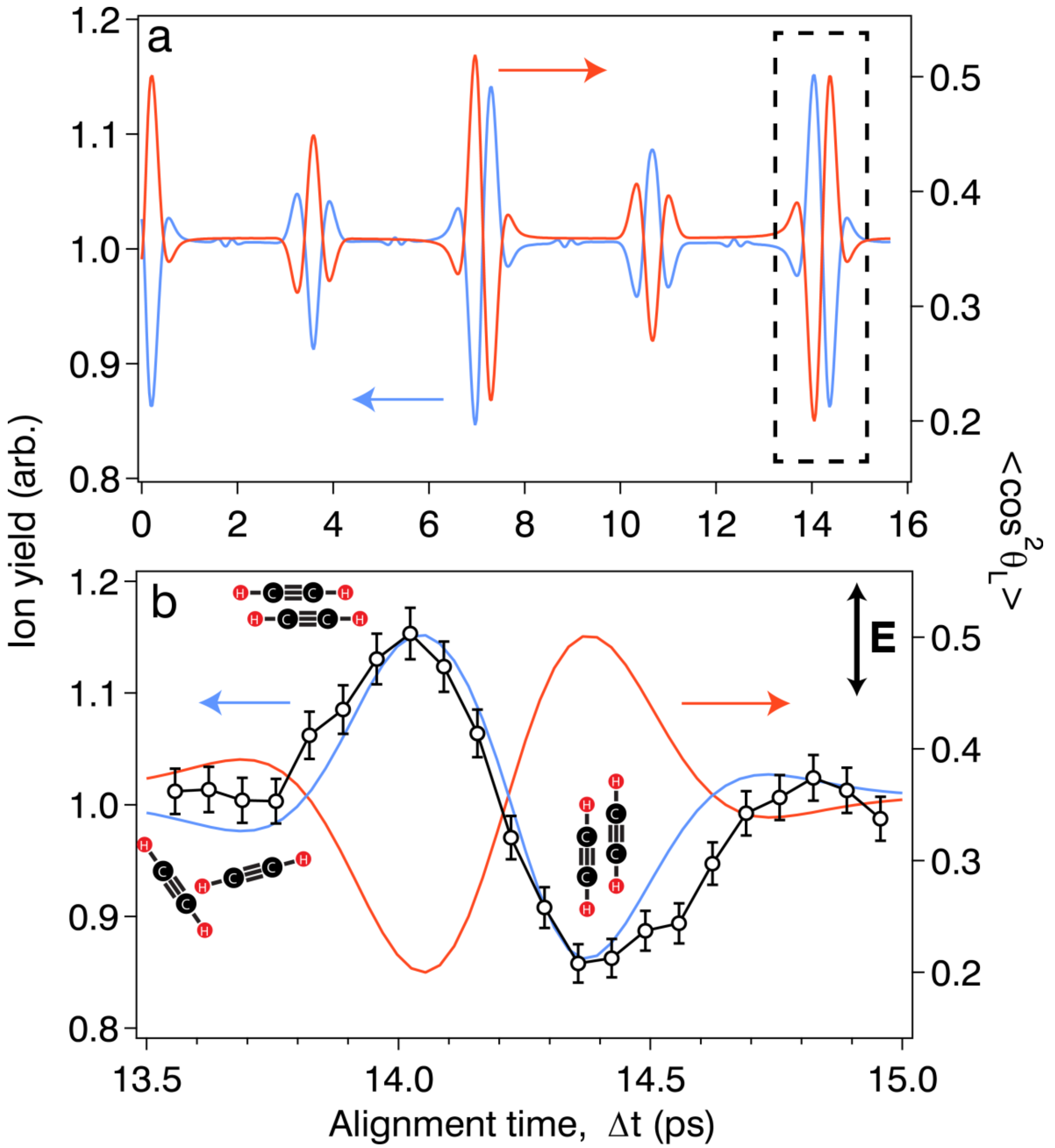

Supplementary Figure 1: a) The simulated ion signal (blue) and degree of alignment (red) as a function of the alignment time. The full revival is present near 14 ps and is outlined by a black dashed box. b) A zoomed in view of the full revival where the experimental ion signal has been included (black curve). The positions of molecular unalignment, alignment and anti-alignment with respect to the laser polaristion (top right corner) are indicated. An estimated maximum value of $\langle \cos^2 \theta_L \rangle = 0.5$ is achieved at 14.40 ps. The s.d. error bars are derived from Poissonian statistics.

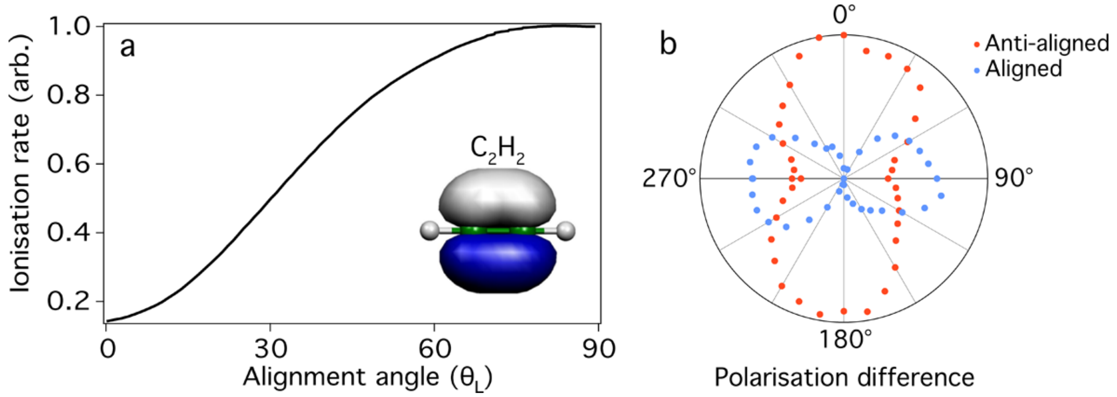

Supplementary Figure 2: a) The normalised C_2H_2 ionisation rate versus alignment angle for the SFA. The laser intensity is 1 x 10^{14} W cm^{-2} and the inset shows the C_2H_2 HOMO. The ionisation rate is expected to be maximal near 90°. b) Experimental confirmation of the expected ionisation rate as a function of the alignment angle. The pump polarisation was varied at the positions of alignment (blue) and anti-alignment (red).

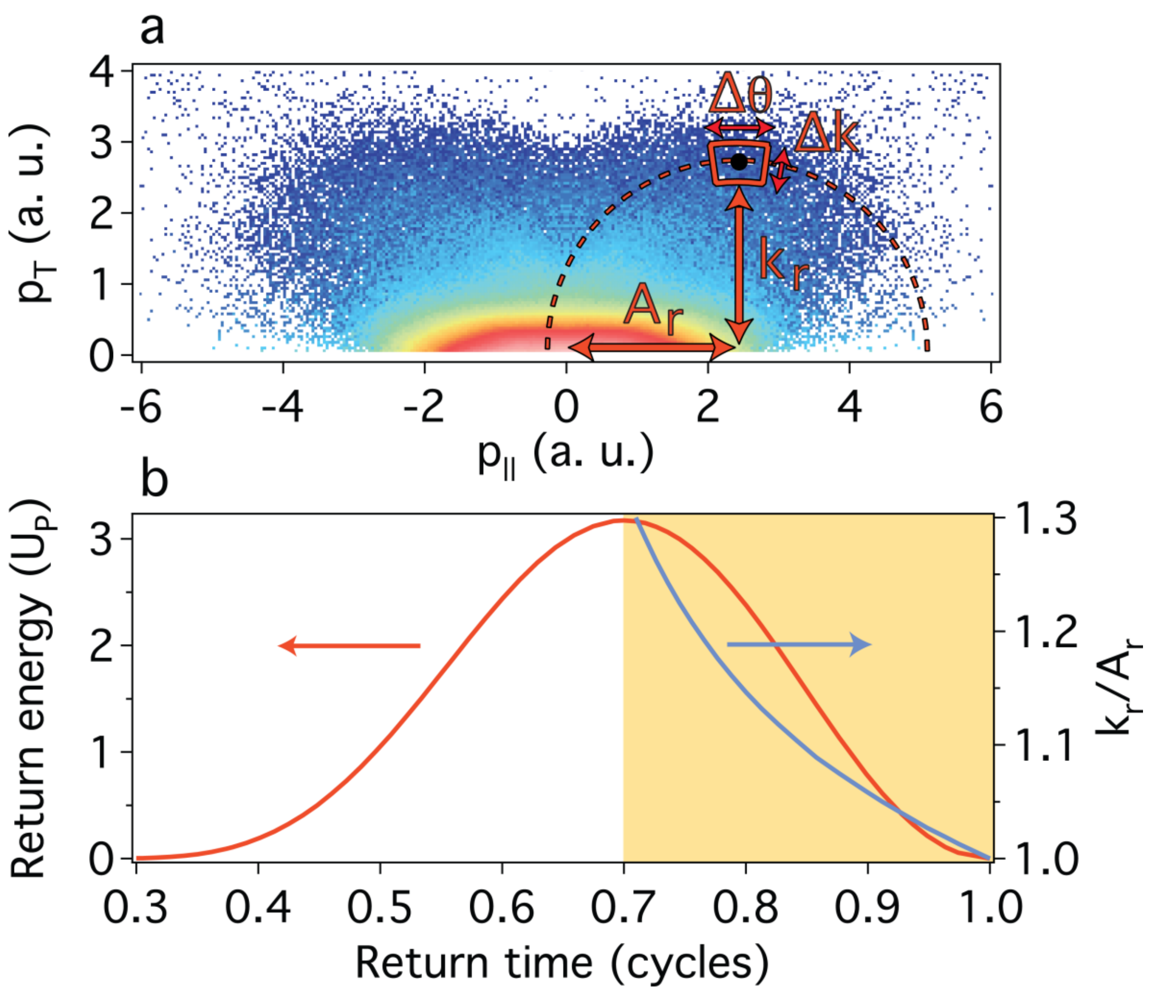

Supplementary Figure 3: a) The experimental DCS is extracted from the electron momentum distribution as outlined in the text. The integration range is increased to reach an adequate signal-to-noise level. This example shows an exaggerated view of the extraction of the DCS data point at a scattering angle of 90°. b) The values of k_r and A_r can be calculated classically and the ratio of the two is shown for the long trajectories (orange shading).

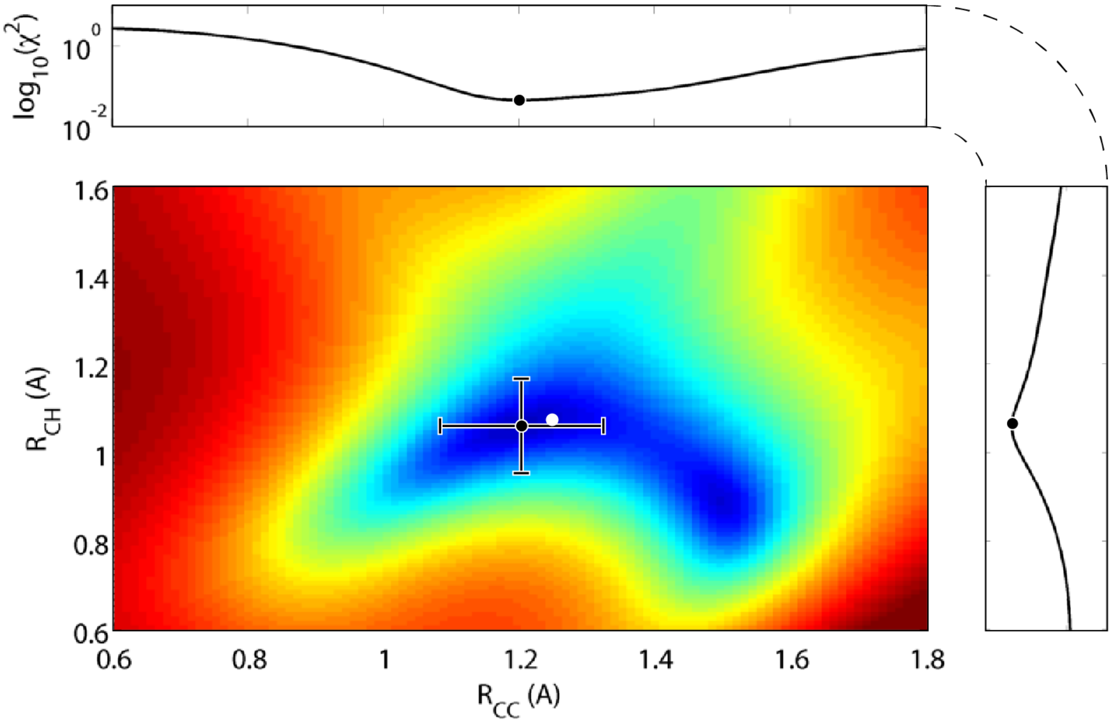

Supplementary Figure 4: A logarithimcally scaled map of the multiple bond length fitting routine for a particular electron energy and molecular alignment. The known bond lengths are indicated by the white dot while the position of best fit ($\chi^2_{\min}$) is indicated by the black dot. The top and right panels show lineouts through $\chi^2_{\min}$ with the minimum positions indicated. The error bars are derived from the extremes of a contour at $1.1 \cdot \chi^2_{\min}$ for each fitting routine. The extremes are typically 5-10% away from $\chi^2_{\min}$, we therefore conservatively used an estimate of 10% for all extracted bond lengths.

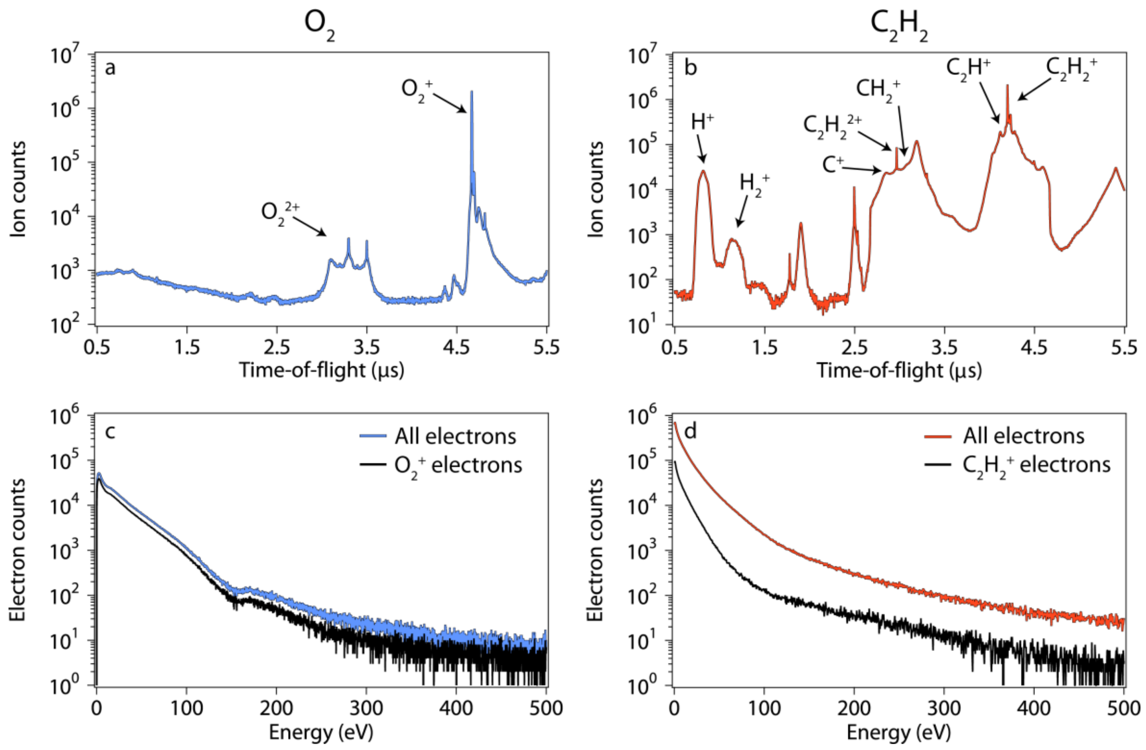

Supplementary Figure 5: a) The ion time-of-flight (TOF) spectrum measured after the ionisation of O_2 with our 3.1 μm OPCPA source. The two main features are indicated and are due to single and double ionisation. The number of double ions is three orders of magnitude lower than the number of single ions. b) The equivalent ion TOF after ionisation of C_2H_2 molecules. The results of many other fragmentation processes can clearly be observed and each of these fragments has associated electrons. c) The electron spectrum corresponding to the O_2^+ ion only (black curve) is of a similar magnitude to the total number of detected electrons (blue curve). d) The number of detected electrons from the $C_2H_2^+$ ion only (black curve) are an order of magnitude lower than the total number of detected electrons (red curve). These unwanted electrons can contaminate LIED experiments and make structural retrieval impossible for polyatomic molecules when coincidence detection is not used.

Supplementary Notes

Supplementary Note 1

Impulsive laser alignment. The 98 fs, 1.7 μm output of the OPCPA is used to impulsively align the C_2H_2 by focusing it into the output of the supersonically expanded gas jet, which has an estimated rotational temperature of 90 K. The molecules are either aligned along the polarisation direction of the laser field or anti-aligned, meaning perpendicular to the polarisation. As this pulse leaves the interaction region, the molecules periodically align or anti-align at intervals that are governed by the rotational constant of the molecule[31]. We measure the degree of alignment by monitoring the ion yield as a function of the delay between the alignment pulse and the intense 3.1 μm pulse. Supplementary Fig. 1b presents the experimental results of this delay scan (black curve) where the positions of molecular alignment and anti-alignment can be observed at time delays of 14.05 ps and 14.40 ps, respectively. The polarisation directions of both the pump and probe radiation were parallel, as indicated in the top right corner.

To simulate the molecular alignment distribution, we solve the time dependent Schrödinger equation (TDSE) for the molecule in the pump laser pulse while treating it as a rigid rotor[1] with a rotational constant of $B = 1.177\ \mathrm{cm}^{-1}$ [2], polarizability of $\alpha_{\parallel} = 31.634$ a.u. (atomic units), and $\alpha_{\perp} = 19.445$ a.u. The polarizability was obtained from calculations using *ab initio* quantum chemistry GAUSSIAN code[3] (see below). The polarizability anisotropy is $\Delta\alpha = \alpha_{\parallel} - \alpha_{\perp} = 12.189$ a.u., which is quite close to the value of $\Delta\alpha = 1.74\ \text{Å}^3$ (11.74 a.u.)[4]. We assume a Boltzmann distribution of the rotational levels at the initial time. Due to nuclear spin statistics, the ratio between odd and even rotational angular momentum J is taken as 3:1. The time-dependent alignment distribution $\rho(\theta_L, t)$ can be calculated as

$$\rho(\theta_L, t) = \sum_{JM} \omega_{JM} \left| \Psi_{JM}(\theta_L, \phi_L, t) \right|^2, \qquad (1)$$

where $\Psi_{JM}(\theta_L, \phi_L, t)$ is the molecular wavefunction, evolved from initial $\Psi_{JM}(\theta_L, \phi_L, t = -\infty) = \left| JM \right\rangle$ and ω_{JM} is the weight according to the Boltzmann distribution. The degree of alignment can be estimated by $\left\langle \cos^2 \theta_L \right\rangle$ and is shown in Supplementary Fig. 1a (red curve). To reach this level of agreement we used the following parameters: a pump pulse intensity of 2.0 x 10^{13} W cm^{-2}, a wavelength of 1.7 μm, a

pulse duration of 90 fs (FWHM), and rotational temperature of 90 K. These parameters have been adjusted to match the experimental time delay measurement of the ion signal (blue curve) and are also close to the experimental estimates. Near the full revival at 14.40 ps a maximum alignment of $\left\langle \cos^2 \theta_L \right\rangle \approx 0.50$ is achieved. The full range of the simulated ion signal and degree of alignment is presented in Supplementary Fig. 1a. The dashed black box represents the region of interest for the data presented in Supplementary Fig. 1b. The ion signal and the alignment degree are out of phase with each other as expected (see below) and as has been shown previously in high harmonic generation measurements[5].

In our simulation of the ion signal we use the strong-field approximation (SFA)[6-8] to calculate ionization rate from aligned and anti-aligned C_2H_2. The highest occupied molecular orbital (HOMO) is calculated using the ab initio quantum chemistry GAUSSIAN code[3] within the density functional theory (DFT) with Becke's three-parameter Lee-Yang-Parr hybrid functional (B3LYP) and the augmented correlation-consistent polarized valence triple-zeta (aug-cc-pVTZ) basis set. A typical ionisation rate versus alignment angle calculated using the SFA is shown in Supplementary Fig. 2a for a laser intensity of 1.0 x 10^{13} W cm^{-2}. Note that the ionisation probability is maximal when molecules are anti-aligned, i.e., when the molecular axis is perpendicular to the polarisation direction of the ionising pulse. This is understood by the molecular tunneling ionisation theory[9], which states that tunnel ionisation rate is proportional to the electron density in the direction of laser polarisation direction. Since the outermost electron of acetylene has π_μ symmetry, ionisation is maximal when the molecule is aligned at 90° with respect to the polarisation direction of the ionising radiation. The ionisation rate therefore minimises near 0°, which reflects the nodal line of the HOMO along the molecular axis.

Experimental confirmation of this behaviour is presented in Supplementary Fig. 2b. The ion signal was measured as a function of the difference in the polarisation directions of the pump and probe pulses for the delay times corresponding to alignment (blue) and anti-alignment (red), as indicated in Supplementary Fig. 1b. When the pump and probe pulses have the same polarisation the anti-aligned signal maximises and the aligned signal minimises, hence confirming the predicted behaviour in Supplementary Fig. 2a.

Supplementary Note 2

Quantitative rescattering theory. According to the Quantitative Rescattering (QRS) theory, the photoelectron momentum distribution $D(k,\theta)$ is related to the elastic DCS by[10,11,12]

$$D(k,\theta) = W(k_r)\sigma(k_r,\theta_r), \qquad (2)$$

where $W(k_r)$ is the returning electron wave packet. Since the returning electron scatters from the target in the laser field at time t_r, it therefore gains an additional momentum shift $-A(t_r) \equiv -A_r$ from the laser. Assuming that the laser is polarized along *y*-axis, the relation between the scattered electron momentum k_r and the detected photoelectron momentum k is given by

$$k_{\parallel} = k\cos\theta = -A_r \pm k_r \cos\theta_r,$$

$$k_{\perp} = k\sin\theta = k_r \sin\theta_r, \qquad (3)$$

where plus (minus) sign refers to electron returning back to the target along the +y (-y) direction. It was found that long trajectories dominate over short trajectories due to the higher ionisation probability. Thus we need to calculate the momentum shift A_r, corresponding to any k_r for long trajectories only. This approach was also taken in Ref. 13. Alternatively, one can use a phenomenological relationship $A_r = k_r/1.26$ [12], which has been found to be quite adequate even for electron returning energies slightly above the classical value (i.e. return energies above $3.17U_P$).

Equations 1-3 above were written for atomic targets. Similar equations hold for fixed-in-space molecules. Since the pump and probe lasers polarizations are kept parallel in our experiments, cylindrical symmetry is preserved. Taking into account that the returning wave packet is proportional to the ionization rate $N(\Omega_L)$, we can write

$$D(k,\theta,\Omega_L) = W(k_r,\Omega_L)\sigma(k_r,\theta_r,\Omega_L) = \widetilde{W}(k_r)N(\Omega_L)\sigma(k_r,\theta_r,\Omega_L) \qquad (4)$$

where Ω_L is the alignment angle of the molecule with respect to the laboratory frame. The observed photoelectron spectrum is obtained by the incoherent sum of the contributions from all molecules in the interaction region[42]. Assuming that molecular alignment distribution is described by $\rho(\Omega_L)$, the total signal can be written as

$$\overline{D}(k,\theta) \propto \widetilde{W}(k_{\mathrm{r}})\int d\Omega_L \rho(\Omega_L) N(\Omega_L)\sigma(k_{\mathrm{r}},\theta_{\mathrm{r}},\Omega_L). \quad (5)$$

It is clear that Eq. 5 together with Eqs. 2-3 allow us to relate the observed photoelectron momentum spectrum with DCSs weighted by ionization rates and the alignment distribution,

$$\frac{\overline{D}(k,\theta)}{\widetilde{W}(k_{\mathrm{r}})} \propto \int d\Omega_L \rho(\Omega_L) N(\Omega_L)\sigma(k_{\mathrm{r}},\theta_{\mathrm{r}},\Omega_L) \equiv I_{\mathrm{tot}}(k_{\mathrm{r}},\theta_r). \quad (6)$$

In other words, a weighted DCS can be measured in LIED experiments up to an overall scaling factor.

Supplementary Note 3

Experimental data extraction. The extraction of DCSs from experimentally measured electron momentum distributions is based upon the QRS theory outlined above. To extract a DCS we use the coincidence conditions of the ReMi to extract the momentum distribution from the electrons corresponding to singly ionised C_2H_2 only. This means that electrons corresponding to multiple ionisation, fragmentation and other processes are ignored. The importance of this condition is highlighted in Fig. 3b in the main text where the MCF obtained when using only the singly ionised C_2H_2 electrons (black) is compared to the MCF obtained when this condition is not imposed (red). The data from all electrons clearly does not follow the fitted curve. Secondly, the full three dimensional momentum distribution is integrated around the azimuthal angle in cylindrical co-ordinates before being converted to Cartesian co-ordinates with the application of the appropriate Jacobian ($J = 1/k_{\perp}$). This gives the momentum distribution as a function of the longitudinal ($k_{\parallel}$) and transverse ($k_{\perp}$) momenta, as shown in Supplementary Fig. 3a. It is from this data representation that we extract the accurate DCSs.

The angularly resolved DCS is obtained by sweeping the scattering angle around the circumference of a circle with radius $k_{\mathrm{r}} = \sqrt{2E}$, where E is the electron return energy, that has been shifted in the laser polarisation direction by the vector potential at the time of rescattering A_{r} (see Supplementary Fig. 3a). The $k_{\mathrm{r}} / A_{\mathrm{r}}$ ratio can be calculated as a function of the return time using classical trajectory analysis[12] and is presented in Supplementary Fig. 3b. The well-known electron return energy curve (red) shows both the short trajectories (t < 0.7 cycles) and the long trajectories (t > 0.7 cycles). The long trajectories are indicated by the orange shaded region. As described above, only long trajectories are considered in this

analysis so the k_r / A_r ratio (blue curve) is only presented in this range.

After finding the appropriate values of k_r and A_r, obtaining the DCS is merely a matter of counting the number of electrons found at each scattering angle (θ_r). In reality, a certain signal-to-noise level must be reached in order to obtain accurate DCSs. We solved this problem by increasing the integration range in the direction of the rescattered electron (Δk) and angle ($\Delta\theta$) for each scattering angle, as presented in Supplementary Fig. 3a for $\theta_r = 90\,°$. Typical values are $\Delta k = 0.15 k_r$ and an angular width of a few degrees. Once the DCS is obtained, the MCF can be calculated according to Eq. 10.

Supplementary Note 4

Independent atom model. In this paper we use the independent-atom model (IAM) to calculate the DCS[13,15]. This approach has been found to be adequate for relatively large scattering angles ($\theta_r \geq 40\,°$)[16]. Within the IAM, the DCS can be expressed as

$$\sigma(k_r, \theta_r, \Omega_L) = \sum_{i,j} f_i, f_j^* e^{i\vec{q}.\vec{R}_{ij}} , \qquad (7)$$

where $f_i \equiv f_i(k_r, \theta_r)$ is the scattering amplitude from the i-th atom in the molecule, $\vec{R}_{ij} = \vec{R}_i - \vec{R}_j$ and $\vec{q} = \vec{k}_r - \vec{k}_{r0}$. Combining Eq 6 with Eq. 7, the weighted DCS can be approximated as

$$I_{tot}(k_r, \theta_r) = \sum_i |f_i|^2 \int d\Omega_L \rho(\Omega_L) N(\Omega_L) + \sum_{i \neq j} f_i f_j^* \int d\Omega_L \rho(\Omega_L) N(\Omega_L) e^{i\vec{q}.\vec{R}_{ij}} . \qquad (8)$$

This equation is merely a straightforward extension of Eq. 7 by Xu *et al.*[15] for the case of aligned molecules. Following Ref. 15, we define a molecular contrast factor (MCF) as the ratio of the molecular interference term (the second term of Eq. 8) and the atomic term (the first term of Eq. 8)

$$\gamma(k_r, \theta_r) \equiv \frac{I_{tot} - I_\alpha}{I_\alpha} = \frac{\sum_{i \neq j} f_i f_j^* \int d\Omega_L \rho(\Omega_L) N(\Omega_L) e^{i\vec{q}.\vec{R}_{ij}}}{\sum_i |f_i|^2 \int d\Omega_L \rho(\Omega_L) N(\Omega_L)} . \qquad (9)$$

Since the experimental DCS can be measured up to an overall factor for a fixed scattering energy, the experimental MCF can be defined as

$$\gamma^e(k_r, \theta_r) \equiv \frac{I_{tot}^e - I_\alpha}{I_\alpha} = \frac{\beta D^e - I_\alpha}{I_\alpha} , \qquad (10)$$

where β can be found by best fit to the theoretical MCF and D^{e} is the experimentally measured DCS.

Supplementary Note 5

Bond length extraction. Molecular structure parameters (symbolically denoted in the following as $\vec{R}_{\mathrm{a}}$) can be retrieved in LIED experiments from the best chi-square fit of the experimental MCF to the theoretical one

$$\chi^2(\vec{R}_{\mathrm{a}}) = \sum_{ij} (\gamma^{\mathrm{e}}(k_i, \theta_j) - \gamma(k_i, \theta_j; \vec{R}_{\mathrm{a}}))^2 . \qquad (11)$$

Here for simplicity we have omitted subscript r in k_{r} and θ_{r}. $\{i, j\}$ gives the indices of grid points of scattering energies (or momenta) and scattering angles, respectively, available in the experiment. Thus Eq. 11 gives the best overall fit for the available range of scattering energy and angle. This procedure can be called 2D-fitting. Note that in Ref. 13 the fitting was carried out for fixed scattering energies. Their 1D-fitting procedure corresponds to limiting the double summation in Eq. 11 to a single summation over *j*. Clearly, one can also choose to fix scattering angle and vary the scattering energy, which leads to summation over *i* only.

Supplementary Fig. 4 presents the results of fitting the two bond lengths for an electron energy of 65 eV over a large parameter range. The colour scale represents $\log(\chi^2)$ as a function of the C-C and C-H bond lengths. The minimum position ($\chi^2_{\min}$) is indicated by the white dot and the known cation equilibrium position is indicated by the black dot. The panels on the right and left show lineouts that go through $\chi^2_{\min}$. In general, the fits have individual error bars on the order of 10%. The vertical and horizontal lines in Supplementary Fig. 4 represent these ±10% error bars, which overlap with the known values in this example.

Supplementary references

1. Stapelfeldt, H. & Seideman, T. Aligning molecules with strong laser pulses. *Rev. Mod. Phys.* **75**, 543-557 (2003).

2. Herzberg, G. *Electronic spectra and electronic structure of polyatomic molecules.* Van Nostrand, New York (1966).

3. Frisch, M. J. *et al.*, GAUSSIAN 03, revision C.02, (Gaussian Inc., Pittsburgh, PA, 2003).

4. Rizzo, A. & Rahman, N. The Linear and Nonlinear Susceptibilities of Acetylene Relevant for High Order Harmonic Generation. *Laser Phys*. **9**, 416-421 (1999).

5. Torres, R. *et al.* Probing Orbital Structure of Polyatomic Molecules by High-Order Harmonic Generation. *Phys. Rev. Lett*. **98**, 203007 (2007).

6. Keldysh, L. V. Ionization in the field of a strong electromagnetic wave. *Sov. Phys. JETP* **20** 1307-1314 (1965).

7. Lewenstein, M., Balcou, Ph., Ivanov, M. Yu., L'Huillier, A. & Corkum, P. B. Theory of high-harmonic generation by low-frequency laser fields. *Phys. Rev. Lett.* **49**, 2117 (1994).

8. Le, A. T., Lucchese, R. R., Tonzani, S., Morishita, T. and Lin, C. D. Quantitative rescattering theory for high-order harmonic generation from molecules. *Phys. Rev. A* **80**, 013401 (2009).

9. Tong, X. M., Zhao, Z. X. & Lin, C. D. Theory of molecular tunneling ionization. *Phys. Rev. Lett.* **66**, 033402 (2002).

10. Lin, C. D., Le, A. T., Chen, Z., Morishita, T. & Lucchese, R. Strong-field rescattering physics-self-imaging of a molecule by its own electrons. *J. Phys. B* **43**, 122001 (2010).

11. Morishita, T., Le, A. T., Chen, Z. & Lin, C. D. Accurate Retrieval of Structural Information from Laser-Induced Photoelectron and High-Order Harmonic Spectra by Few-Cycle Laser Pulses. *Phys. Rev. Lett*. **100**, 013903 (2008).

12. Chen, Z., Le, A. T., Morishita, T. & Lin, C. D. Quantitative rescattering theory for laser-induced high-energy plateau photoelectron spectra. *Phys. Rev. A* **79**, 033409 (2009).

13. Blaga, C. I. *et al.* Imaging ultrafast molecular dynamics with laser-induced electron diffraction.

Nature **483**, 194-197 (2012).

14. Madsen, C. B., Mouritzen, A. S., Kjeldsen, T. K. & Madsen, L. B. Effects of orientation and alignment in high-order harmonic generation and above-threshold ionization. *Phys. Rev. A* **76**, 035401 (2007).

15. Xu, J., Chen, Z., Le, A. T. & Lin, C. D. Self-imaging of molecules from diffraction spectra by laser-induced rescattering electrons. *Phys. Rev. A* **82**, 033403 (2010).

16. Xu, J. *et al.* Diffraction using laser-driven broadband electron wave packets. *Nat. Commun.* **5**, 4635 doi: 10.1038/ncomms5635 (2014).
